# Ebola, epidemics, and ethics - what we have learned

**DOI:** 10.1186/1747-5341-9-15

**Published:** 2014-10-24

**Authors:** G Kevin Donovan

**Affiliations:** 1 Pellegrino Center for Clinical Bioethics, Georgetown University Medical School, Bldg. D, Rm 236, 4000 Reservoir Road, N.W., Washington, DC 20007-2197, USA

**Keywords:** Ebola, Epidemic, Ethics, Experimental treatments, Global health

## Abstract

The current Ebola epidemic has presented challenges both medical and ethical. Although we have known epidemics of untreatable diseases in the past, this particular one may be unique in the intensity and rapidity of its spread, as well as ethical challenges that it has created, exacerbated by its geographic location. We will look at the infectious agent and the epidemic it is causing, in order to understand the ethical problems that have arisen.

## Introduction

The current Ebola epidemic has presented challenges both medical and ethical. Although we have known epidemics of untreatable diseases in the past, this particular one may be unique in the intensity and rapidity of its spread, as well as ethical challenges that it has created, exacerbated by its geographic location. We will look at the infectious agent and the epidemic it is causing, in order to understand the ethical problems that have arisen.

### The infection

Ebola Virus Disease is caused by one of at least 30 known RNA viruses capable of causing a viral hemorrhagic fever syndrome. The genus *Ebolavirus* is currently classified into five separate subspecies: *Sudan, Zaire, Tai Forest(Ivory Coast), Reston,* and *Bundibugyo ebolavirus*. Wild animals serve as a reservoir and can transmit the virus to humans. It is thought to be endemic in the wild fruit bat. Humans can acquire the virus by eating “bushmeat” such as antelope or monkeys that have been infected. The virus is transmitted in body fluids, primarily blood, saliva, emesis or stool and does not appear to be transmitted through the air. Caregivers of infected patients, and those who prepare them for burial, are at particular risk. Of concern are the recent reports that the virus is showing genetic mutations, even during the current epidemic.

With the African derived Ebola virus infections, there is an incubation period that can last from three days to three weeks. The virus is not spread until the patient becomes symptomatic. Patients develop fever, chills, headache, muscle aches, vomiting and diarrhea. In the later stages they develop a hemorrhagic rash, and bleeding from any mucous membranes. Currently there is no approved specific therapy available for treatment, and no available vaccines for prevention. Intensive general medical support is critical to survival, with IV fluid therapy and control of bleeding disorders. This care must be given with strict attention to barrier isolation from the contaminated fluids from the patient. Such intensive care provides the best likelihood of survival for an infected patient. Current mortality rates in this epidemic are at about 55%, and have ranged as high as 90% in previous epidemics with minimal medical support. Protective barriers for healthcare workers (gloves, gowns, masks and goggles) should be worn for identified Ebola cases. However, identification may be delayed because the early symptoms can resemble malaria, typhoid fever, cholera, and other illnesses which present much more commonly in the geographic region [1].

### The epidemic

The current epidemic of Ebola virus disease is one of two dozen outbreaks since discovery of the virus in 1976. It was identified along the border of Guinea and Sierra Leone last March, subsequently spreading to Liberia, with a handful of cases in Nigeria associated with the arrival of an infected airline passenger [2]. Although EVD in Nigeria seems to be somewhat limited to direct, or secondary contacts of the symptomatic airline passenger, the epidemic continues to proliferate in Guinea, Sierra Leone, and Liberia, with cases now reported in Senegal as well. This has become the largest Ebola outbreak ever, because of the perfect storm of circumstances.

Despite multiple outbreaks in the past, this epidemic is the first to have a major impact on multiple large cities, making it harder to contain than those previous instances in rural areas. It has occurred in the poorest of African countries. They had insufficient infrastructure to mount an adequate response: the few hospitals are largely without adequate equipment, protective barrier clothing, intravenous fluids for the patients, or toilets, many of them with dirt floors.

The cultural and political atmosphere also presents significant challenges for those trying to contain the epidemic. After years of civil war and brutality, there is no trust of the government institutions, nor is there trust of the medical system. Some deny that Ebola is real, even going so far as to claim that there is no epidemic, but the government health workers are killing patients to simulate an epidemic and receive Western funding. Traditional burial practices include hand-washing of the body and large wakes, so families refuse cremation of Ebola victims or even funerals that would allow infection protection such as body bags. Those who are ill stay away from the hospitals for fear of becoming infected with Ebola; those who have Ebola hide to avoid stigmatization. Finally, these countries have among the lowest ratio of physicians and nurses per population in the world, some 200 times less than found in the United States or Canada [3].

Nevertheless, the world took little note that despite these overwhelming challenges West African healthcare workers have been at the front lines since the beginning of the epidemic. They have experienced by far the most infections and the most fatalities among those responding to the epidemic. Only now are we seeing hesitation from local healthcare workers fearful of showing up at hospitals with insufficient gowns and gloves, etc. to protect them. Their fear is not unjustified; this epidemic is averaging a 55% fatality rate with no specific treatments or preventative vaccines yet available.

The current epidemic finally came to widespread attention in the rest of the world when it was announced that two American medical missionaries were infected. Dr. Kent Brantley and Nancy Writebol of the charitable organization SIM were treated with an experimental therapy, Z Mapp, then transferred to an isolation ward at Emory medical school in Atlanta for further supportive therapy, and both subsequently survived. The world and the region had known little of this unapproved medication until their treatment nor did people realize at the time how few doses were available [4]. In fact, Dr. Brantley asked that it be given first to his colleague in case there wasn’t enough for both of them. The scenario was repeated with a Spanish priest who became infected caring for Ebola patients, was brought back in Spain where a Z Mapp dose was given to him, but he died. Following questions of why it wasn’t provided to Africans, it was revealed that it had been available for the doctor who had been leading Sierra Leone’s battle against the Ebola outbreak. The treatment team, from Doctors without Borders and the World Health Organization, agonized through the night and ultimately decided not to try the drug. The doctor, Sheikh Umar Khan, died a few days later, on July 29. Subsequently Liberian doctors received doses of the same medication, reportedly the last available, and one physician has subsequently died.

Meanwhile, the virus has spread, with over 9,000 reported cases (and projected to go as high as a million) and a continued 55% mortality rate. Ineffective measures have been taken, such as restricting air travel and quarantining neighborhoods, as well as more sensible actions such as screening air passengers for symptoms, increasing public health measures with isolation of patients, protective barrier clothing, supportive medical care and diagnostic labs, and safe burial practices. Despite these measures, and an influx of health volunteers and government health workers from other countries, with the support of Doctors Without Borders, the Red Cross, and WHO, the number of cases is projected to increase for many months, rising as high as 20,000. Trials of potential vaccines and curative treatments have begun, but can’t produce any effective and verifiable results for many months or longer.

## Discussion

### Ethical questions

#### ***Should untested experimental treatments be offered in this epidemic?***

We must, of course, consider the pros and cons of giving experimental drugs to people. These are drugs that had never been tested in human subjects before they were given to the sick patients, therefore, the dangerous and adverse effects can neither be known nor safely predicted. It is entirely possible they may be ineffective or even harmful. In America, all FDA approved studies must go through a first stage where the likelihood of harm can first be assessed, followed by subsequent phases to look for additional side effects and evidence of efficacy. So far these therapies have been tested only on a handful of monkeys, and not even the first phase had occurred for human beings.

So far, six people have received one candidate experimental therapy, Z Mapp. Two of them have died. This doesn’t prove that it’s effective, and it doesn’t prove that it’s safe. We don’t know what harms it may do long-term or even short-term, and the guiding principle of the use of any new medicine is “in the first place, do no harm”. Research is designed to answer questions about possible harms and possible effectiveness, but what has been done here is not research, but rather it is scary experimental treatment. It was done because Ebola is a scary deadly disease. But if we were to keep approaching it in this uncontrolled way, we may never know if these therapies are safe and effective, or at least not know until a great deal of harm might occur. The world, especially that part of the world now suffering most from Ebola, desperately needs to know if there can be an effective treatment, as well as an effective prevention in the form of vaccine. We owe it to all possible victims, current and potential, to get this right. There have been examples in the past of untested and under tested therapies being rushed into service, and ultimately doing the patients a disservice. Some of these misadventures occurred on the African continent, leading to a pervasive distrust of Western drug companies using Africans as their experimental “guinea pigs”.

On August 11, 2014, the World Health Organization convened a panel to discuss these ethical issues [3]. Their response was widely reported*: “It is ethical to offer unproven interventions with as yet unknown efficacy and adverse effects, as potential treatment or prevention”.* What received less attention were their qualifiers: the statement applied to *“the particular circumstances of this outbreak,”* and emphasized the *“ethical criteria… including transparency, informed consent, freedom of choice, confidentiality, respect for autonomy and involvement of the community”* which must guide the provision of such interventions. Moreover, *“if and when they are used to treat patients, there is a moral obligation to collect and share all data generated”* and *“there was unanimous agreement that there is a moral duty to also evaluate these interventions in the best possible clinical trials under the circumstances in order to definitively prove their safety and efficacy or provide evidence to stop their utilization”.* This has been my constant position, and it remains so.

#### ***When considering the allocation of scarce resources, the most pressing question is: who should be treated?***

The simple answer is that although the question is ethically very important and complex, it will remain moot until therapies are made available. As we have seen, no tested or approved therapy exists. The remaining stock of experimental therapies is currently exhausted. So, the short answer to “who should be treated?” reverts back to “who can be treated, and how?” To do the most good for the most people affected means to do our best supportive therapy for patients, public health, containment measures for the population, and protective barriers, particularly for the healthcare workers and caretakers on the front line. These are the scarce resources that can be allocated immediately, and must be supplied immediately in order to control this epidemic as soon as possible. Experimental therapies and vaccine trials, as important as they are, cannot be ready for field testing within weeks, but more likely quite a few months or longer, and if successful, even longer until they can be employed on a large scale [5].

#### ***Given that the few doses that were on hand had been used, was it unethical to give them to the American healthcare workers and a Spanish, missionary priest first?***

The answer to this can serve to guide the eventual allocation decisions when such interventions are finally ready. I would contend that an argument can be made that we owe a duty to those workers who have knowingly placed themselves at risk of disease and death in order to serve the needs of others. That duty to first responders is not just to Americans or Europeans, but to the great number of Africans as well, who continue to place themselves in harm’s way, even after record numbers of healthcare workers have been infected and have died in this Ebola epidemic. Their actions reflect the highest altruistic ideals of the medical profession.

Moreover, there is a practical reason to consider treating healthcare workers first. It serves the interests of the majority of patients to keep medical workers and caretakers alive and in the field. It also increases the likelihood that others may be willing to come and help them, if they don’t believe they would be sent to the back of the line or stranded should they succumb to infection. And, finally, healthcare workers might be the ones most likely to accept enrollment in trials of these medications, and understand the requirements of informed consent in the treatment, study, given the widespread misinformation and mistrust regarding Western clinical trials by a large number of Africans.

#### ***Should Americans, Canadians and Europeans who are exposed or infected be brought home while Africans are left behind to die?***

This is one of the most troubling aspects of sending foreign volunteers to help the medical crisis. It seems unjust that Africans, including African healthcare workers, do not have all the life-saving advantages of Westerners. There is clearly inequity here, but it is not the fault of the doctors, nurses and other healthcare workers who came from their countries to help in Africa. It is the intrinsic inequity of the world in which the goods of society are distributed unevenly. If we were to reverse the question, and ask that foreign volunteers should be required to stay and suffer the consequences of an inadequate healthcare system which they are only trying to help, it would perpetuate another kind of injustice. Moreover, the consequence would be to discourage those who are willing to come and help, thereby increasing the disadvantage to those natives of the African countries.

#### If the Ebola virus has been known as the cause of disease since 1976, why is there no preventative vaccine for effective therapy?

Since it first appeared on the scene, there have been nearly 2 dozen outbreaks of Ebola virus disease, *“yet the world was woefully unprepared for the current tragedy, with no licensed vaccines or treatments”*[6]. This in part reflects the arduous process of developing therapeutic medications. Nevertheless, this could have been accomplished in the past four decades. Other forces are clearly at work here. The stark reality is that pharmaceutical companies are in the business of producing therapies that people will pay for, in order to support the staggeringly expensive cost of research. As awful as the Ebola virus is, it has caused far fewer deaths than malaria, tuberculosis, or even diarrheal diseases. Therefore, the sad truth is Ebola represents a small and unrewarding market. Governments often intervene in such situations to help and protect their citizens. In this case, we are dealing with some of the poorest countries on earth, without the funds to provide a minimally decent healthcare system, much less specialized therapies and vaccines. If people were to spread to more developed countries, a greater push for effective interventions might have been demanded, leading to the odious suspicion that the world cared less when the problem is confined to poor African countries. Perhaps the size and rapidity of this epidemic will have led to publicity that pricks the conscience of the developed world, if not to help our brothers, then at least to protect ourselves.

## Conclusion

### What have we learned?

The current outbreak of Ebola Viral Disease has reached epidemic proportions as a result of a perfect storm of conditions: It is a rapidly transmissible, untreatable, and often fatal disease; it has increased exponentially after reaching urban areas; it has spread in the poorest of countries with already inadequate health staffing and facilities. Further, and larger outbreaks of this disease were predictable, but little was done to prevent or prepare for it. These are the truly crucial ethical issues. Secondary but important issues focus on the use of untested and experimental therapies and their just distribution, and further dissemination when additional therapies and vaccine candidate are produced. These issues, coupled to the frightening nature of this disease, served to focus the attention of the developed world on the epidemic in the third world. Now we can best serve the needs of those affected by doing what can help immediately: supplying personal protective barriers, personnel to staff hospitals, laboratories, and cooperation with the governments and health systems of the affected countries. This is the more immediate and immediately achievable goal and should be the prime focus of our ethical and humanitarian concern [7].

The ideas put forth in this editorial, while focused upon the impact of Ebola, call forth other perspectives on the ethical questions, problems, and possible answers relating to the often delicate balance and frequent in balance of scientific, technical, and medical capabilities – and power differential – spawned by differing economies, sociologies and political cultures of developed and developing nations. We anticipate additional contributions to this discussion from other perspectives in the near future.

## Abbreviations

EVD: Ebola Virus Disease; WHO: World Health Organization; FDA: Food and Drug Administration.

## Competing interests

The author declares that he has no competing interests.
